# Supplementary figures and images for: CREB mediates the C. elegans dauer polyphenism through direct and cell-autonomous regulation of TGF-β expression
Source: PLoS Genet. 2021 Jul 14;17(7):e1009678. doi: 10.1371/journal.pgen.1009678 (PMC8312985; doi:10.1371/journal.pgen.1009678)

S1 Fig.

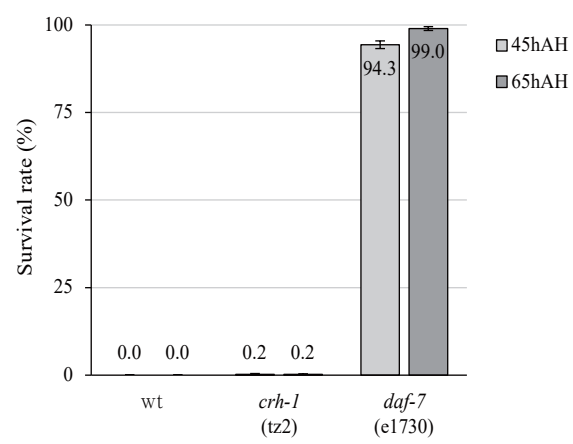

Supplement: S1 Fig — Shown are the survival rate of wild-type, crh-1 mutant and daf-7 mutants animals under 1% sodium dodecyl sulfate (SDS) treatment. Two independent assays with n>50. (PDF) [file pgen.1009678.s001.pdf]

S2 Fig.

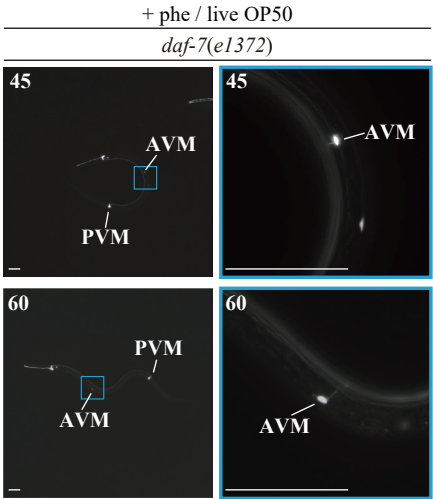

Supplement: S2 Fig — Shown are images of flp-8p::gfp expression in the AVM in daf-7 mutants at 45 and 60 hAH. The boxed regions of the first column are shown on the right columns at higher magnification. hAH, hour after hatching. Scale bars: 50 μm. (PDF) [file pgen.1009678.s002.pdf]

S3 Fig.

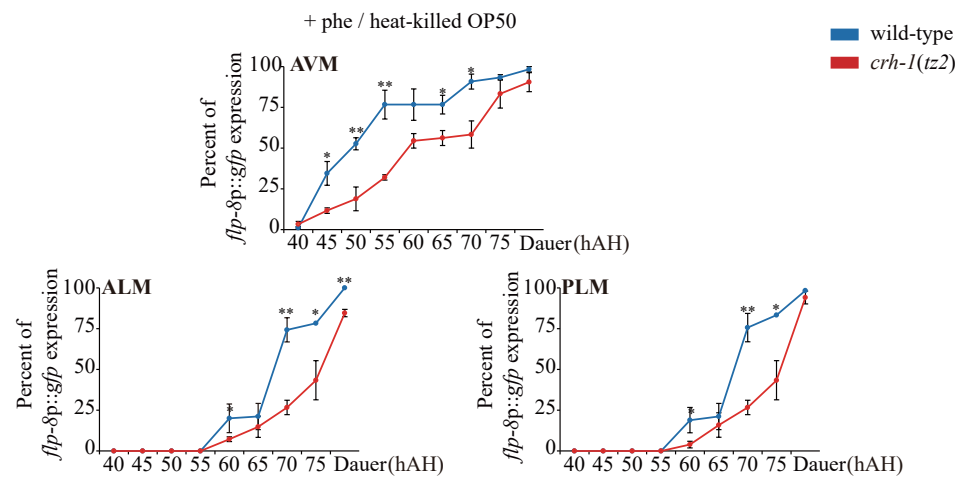

Supplement: S3 Fig — Percent of flp-8p::gfp expression in the AVM, ALM and PLM neurons by wild-type or crh-1 mutant animals when grown in the presence of heat-killed OP50 food and ascr#5 pheromone. n ≥ 30 for each. hAH, hour after hatching. Error bars indicate SEM. * and ** indicate different from wild-type at p < 0.05 and p < 0.01, respectively (student t-test). (PDF) [file pgen.1009678.s003.pdf]

S4 Fig.

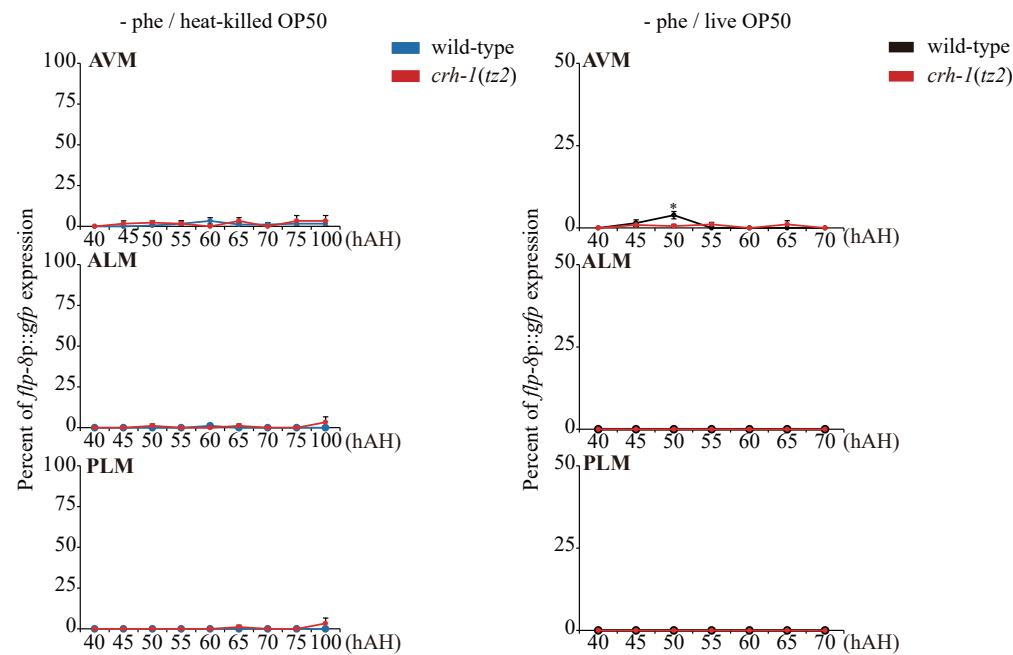

Supplement: S4 Fig — Percent of flp-8p::gfp expression in the AVM, ALM and PLM neurons by wild-type or crh-1 mutant animals when grown in the presence of heat-killed OP50 food (left) or live OP50 (right). n ≥ 30 for each. hAH, hour after hatching. Error bars indicate SEM. * indicates different from wild-type at p < 0.05 (student t-test). (PDF) [file pgen.1009678.s004.pdf]

S5 Fig.

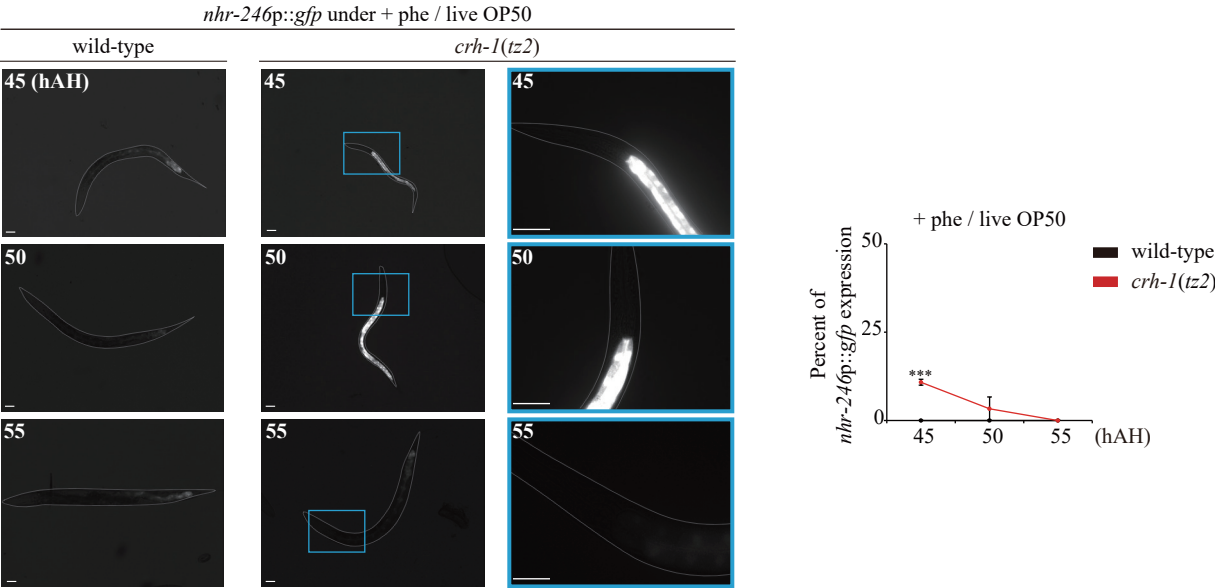

Supplement: S5 Fig — Shown are left images of nhr-246p::gfp expression in the intestine of crh-1 mutants at 45, 50, and 55 hAH. The boxed regions of the second column are shown on the right columns at higher magnification. Scale bars: 50 μm. Shown is the right panel of the percent of nhr-246p::gfp expression in the intestine by wild-type or crh-1 mutant animals when grown in the presence of live OP50 food and ascr#5 pheromone at 45, 50, and 55 hAH. hAH, hour after hatching. n ≥ 20 for each. Error bars indicate SEM. *** indicates different from wild-type at p < 0.001 (student t-test). (PDF) [file pgen.1009678.s005.pdf]

**S6 Fig.**

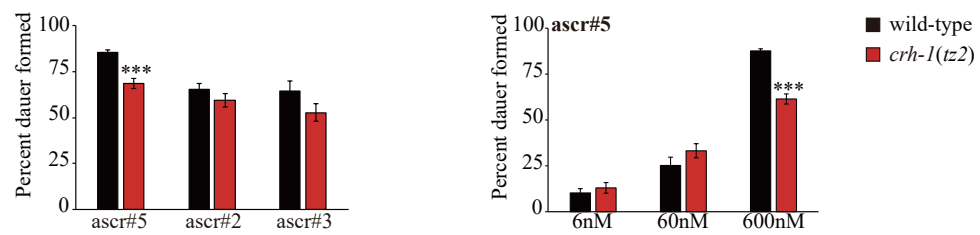

Supplement: S6 Fig — Percent of dauer formed by wild-type or crh-1 mutant animals when grown in the presence of heat-killed OP50 food and ascr#5, ascr#2, or ascr#3 pheromone. (PDF) [file pgen.1009678.s006.pdf]

S7 Fig.

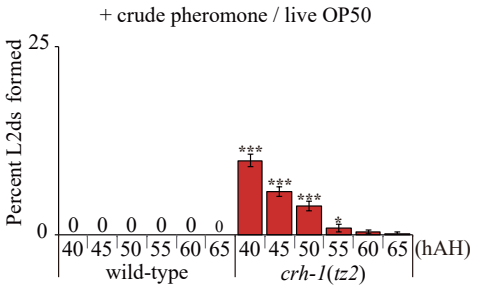

Supplement: S7 Fig — Percent of L2d formed by wild-type or crh-1 mutant animals when grown in the presence of live OP50 food and crude pheromone. N ≥ 5 for each. Error bars indicate SEM. *, **, and *** indicate different from wild-type at p < 0.05, p < 0.01, and p < 0.001, respectively (student t-test). (PDF) [file pgen.1009678.s007.pdf]

**S8 Fig.**

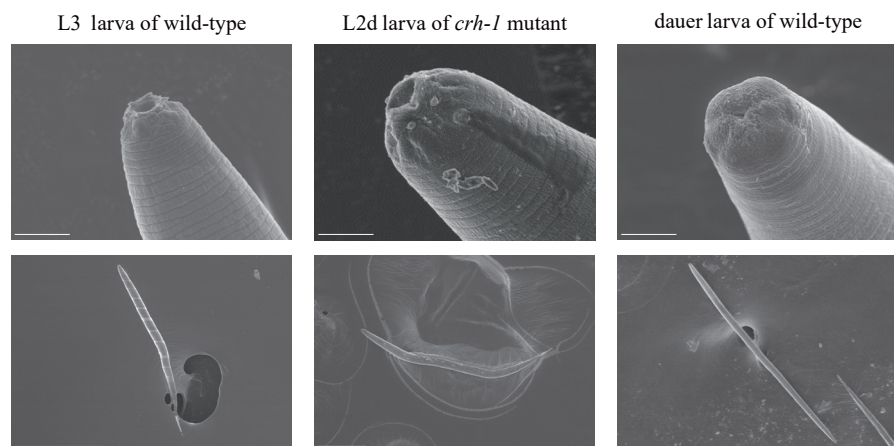

Supplement: S8 Fig — Shown are images of scanning electron microscopy. Scale bar: 5μm (top) and 100μm (bottom). (PDF) [file pgen.1009678.s008.pdf]

S9 Fig.

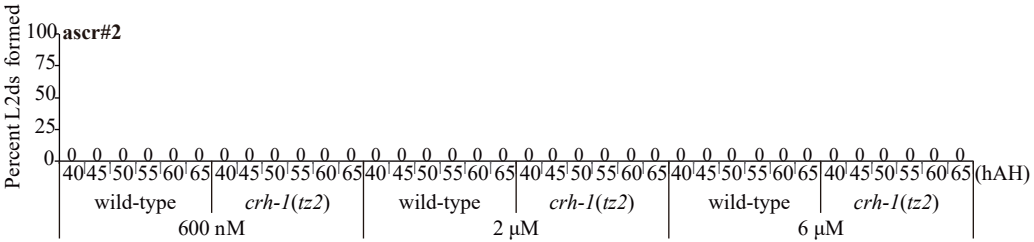

Supplement: S9 Fig — Percent of L2d formed by wild-type or crh-1 mutant animals when grown in the presence of live OP50 food and three different concentrations of ascr#2 pheromone. N ≥ 5 for each. (PDF) [file pgen.1009678.s009.pdf]

S10 Fig.

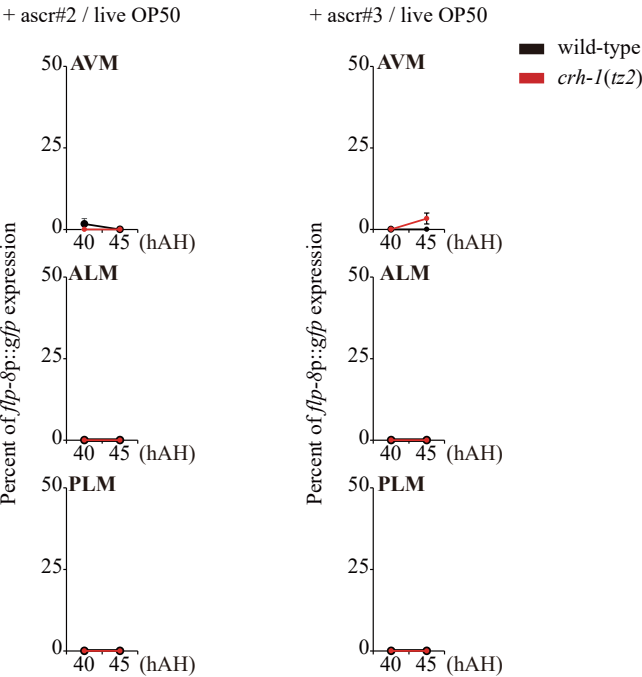

Supplement: S10 Fig — Percent of flp-8p::gfp expression in the AVM, ALM and PLM neurons by wild-type or crh-1 mutant animals when grown in the presence of live OP50 and ascr#2 or ascr#3. n ≥ 30 for each. hAH, hour after hatching. Error bars indicate SEM. (PDF) [file pgen.1009678.s010.pdf]

**S11 Fig.**

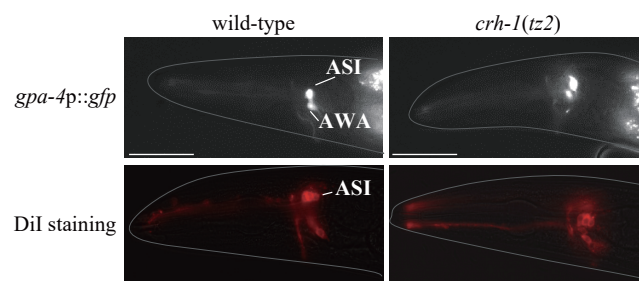

Supplement: S11 Fig — Shown are images of wild-type or crh-1 mutant animals expressing gpa-4p::gfp transgene or stained with DiI. Scale bar: 10 μm. (PDF) [file pgen.1009678.s011.pdf]

S12 Fig.

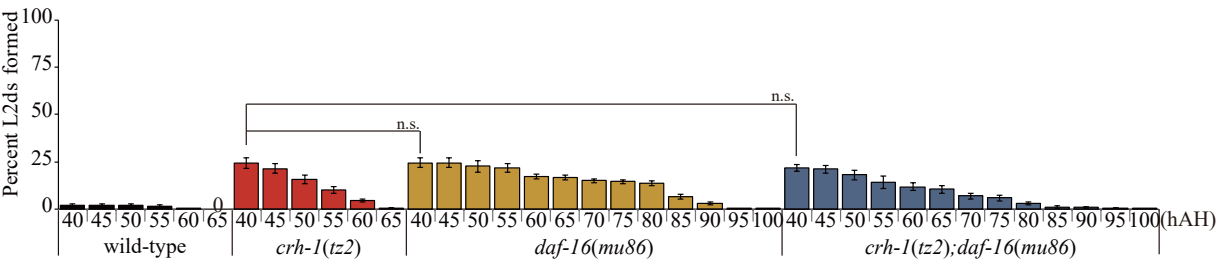

Supplement: S12 Fig — Percent of L2d formed by animals of the indicated genotypes when grown in the presence of live OP50 food and ascr#5 pheromone. N ≥ 4 for each. Error bars indicate SEM. N.S. not significantly different (one-way ANOVA with Bonferroni’s post hoc tests). (PDF) [file pgen.1009678.s012.pdf]

**S13 Fig.**

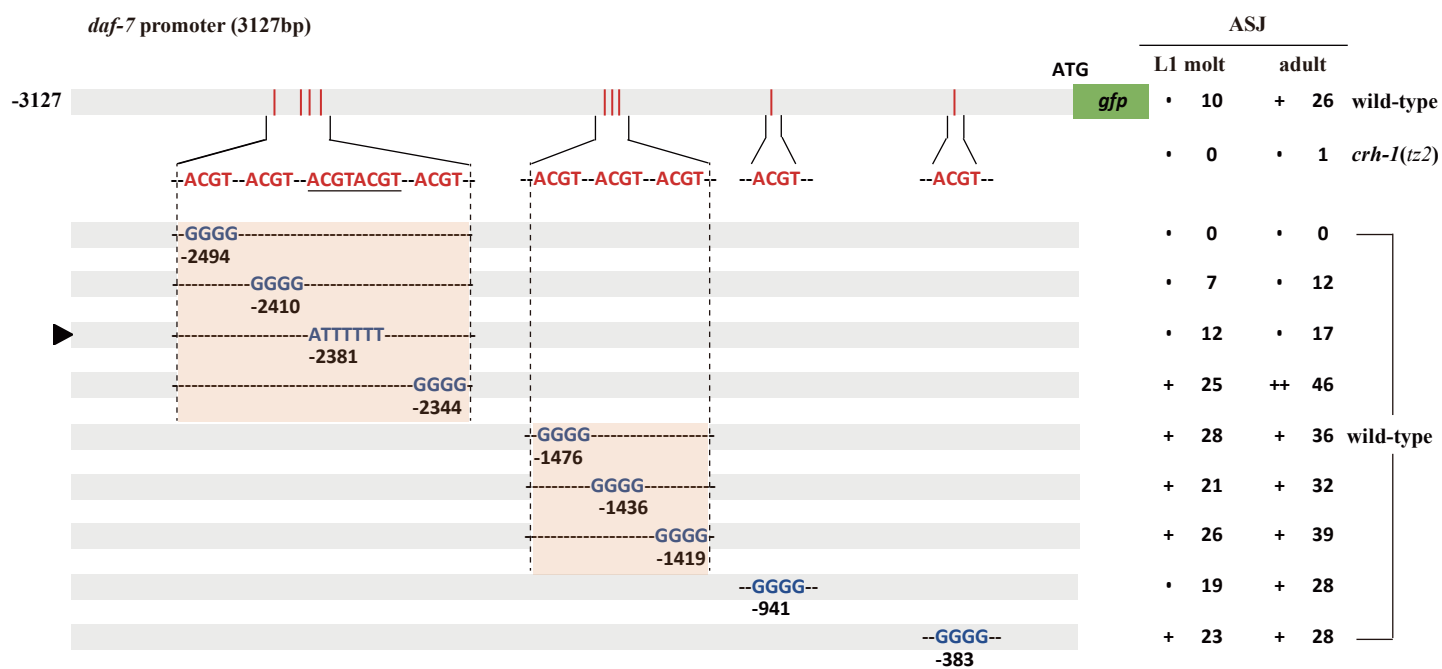

Supplement: S13 Fig — The percentage of transgenic animals expressing daf-7p::gfp reporter construct in the ASJ neurons is shown. GFP fluorescence was observed either in wild-type or crh-1 mutant animals. The Strength of GFP expression is indicated by the number of + symbols. Wild-type nucleotides are indicated in red, mutated nucleotides in blue. An arrowhead indicates daf-7p-mutated CRE promoter. At least two independent extrachromosomal lines for each construct were examined. n ≥ 30 for each. (PDF) [file pgen.1009678.s013.PDF]
