# Supplementary material for: CREB mediates the C. elegans dauer polyphenism through direct and cell-autonomous regulation of TGF-β expression
Source: PLoS Genet. 2021 Jul 14;17(7):e1009678. doi: 10.1371/journal.pgen.1009678 (PMC8312985; doi:10.1371/journal.pgen.1009678)
Supplement: S1 Table — (DOCX) [file pgen.1009678.s014.docx]

**S1 Table. List of strains used in this work.**

| Strain | Genotype | Source and/or  parent strains^a^ | Relevant Figures |
| --- | --- | --- | --- |
| WT | N2 (Bristol) | CGC |  |
| YT17 | *crh-1*(*tz2*) III | CGC |  |
| KHK1401 | Ex[*flp-8*p::*gfp*] |  | 1B-C,2A-B, 3E, 5B, S3, S4, S10 |
| KHK1402 | *crh-1*(*tz2*) III ;Ex[*flp-8*p::*gfp*] | crossed from KHK1401 | 2A-B, 3E, 5B, S3, S4, S10 |
| PY8244 | *srg-36 srg-37(kyIR88)* X |  | 2E |
| KHK1364 | *crh-1*(*tz2*) III ; *srg-36 srg-37(kyIR88)* X |  | 2E |
| PY6561 | *srbc-64(tm1946); srbc-66(tm2943)* X |  | 2E |
| KHK1365 | *crh-1*(*tz2*) III ; *srbc-64(tm1946); srbc-66(tm2943)* X |  | 2E |
| KHK1423 | Ex[*crh-1*p1::*gfp* *unc-122*p::*dsRed*] line1 |  | 3A-B |
| KHK1424 | Ex[*crh-1*p1::*gfp* *unc-122*p::*dsRed*] line2 |  | 3A-B |
| KHK1425 | Ex[2411bp *crh-1*p1 (digested by HindⅢ&PsiⅠ)::*gfp* *unc-122*p::*dsRed*] line1 |  | 3A |
| KHK1426 | Ex[2411bp *crh-1*p1 (digested by HindⅢ&PsiⅠ)::*gfp* *unc-122*p::*dsRed*] line2 |  | 3A |
| KHK1427 | Ex[1094bp *crh-1*p1 (digested by HindⅢ&SnaBⅠ)::*gfp* *unc-122*p::*dsRed*] line1 |  | 3A |
| KHK1428 | Ex[1094bp *crh-1*p1 (digested by HindⅢ&SnaBⅠ)::*gfp* *unc-122*p::*dsRed*] line2 |  | 3A |
| KHK1429 | Ex[*crh-1*p2::*gfp* *unc-122*p::*dsRed*] line1 |  | 3A-B |
| KHK1430 | Ex[*crh-1*p2::*gfp* *unc-122*p::*dsRed*] line2 |  | 3A-B |
| KHK1328 | *crh-1*(*tz2*) III; *Ex[crh-1*p1::*crh-1*cDNA *unc-122*p::*dsRed]* line1 |  | 3C |
| KHK1329 | *crh-1*(*tz2*) III; *Ex[crh-1*p1::*crh-1*cDNA *unc-122*p::*dsRed]* line2 |  | 3C |
| KHK1360 | *crh-1*(*tz2*) III; *Ex[crh-1*p2::*crh-1*cDNA *unc-122*p::*dsRed]* line1 |  | 3C |
| KHK1361 | *crh-1*(*tz2*) III; *Ex[crh-1*p2::*crh-1*cDNA *unc-122*p::*dsRed]* line2 |  | 3C |
| KHK1330 | *crh-1*(*tz2*) III; *Ex[srg-47*p::*crh-1*cDNA *unc-122*p::*dsRed]* line1 |  | 3D |
| KHK1331 | *crh-1*(*tz2*) III; *Ex[srg-47*p::*crh-1*cDNA *unc-122*p::*dsRed]* line2 |  | 3D |
| KHK1338 | *crh-1*(*tz2*) III; *Ex[ttx-1*p::*crh-1*cDNA *unc-122*p::*dsRed]* line1 |  | 3D |
| KHK1339 | *crh-1*(*tz2*) III; *Ex[ttx-1*p::*crh-1*cDNA *unc-122*p::*dsRed]* line2 |  | 3D |
| KHK1334 | *crh-1*(*tz2*) III; *Ex[ceh-36*Δp::*crh-1*cDNA *unc-122*p::*dsRed]* line1 |  | 3D |
| KHK1335 | *crh-1*(*tz2*) III; *Ex[ceh-36*Δp::*crh-1*cDNA *unc-122*p::*dsRed]* line2 |  | 3D |
| KHK1535 | KHK1402 Ex[*srg-47p*::*crh-1*cDNA*; unc-122*::*dsRed*] line1 |  | 3E |
| FK181 | *ksIs2*[*daf-7*p::*gfp* *rol-6*(*su1006*)] | CGC | 4A-D |
| PY6426 | *crh-1*(*tz2*) III; *ksIs2*[*daf-7*p::*gfp* *rol-6*(*su1006*)] |  | 4A-D |
| KHK1434 | PY6426; *Ex[srg-47*p::*crh-1*cDNA *unc-122*p::*dsRed]* line1 |  | 4D |
| KHK1435 | PY6426; *Ex[srg-47*p::*crh-1*cDNA *unc-122*p::*dsRed]* line2 |  | 4D |
| KHK1436 | PY6426; *Ex[srg-47*p::*crh-1*cDNA *unc-122*p::*dsRed]]* line3 |  | 4D |
| CX3596 | *kyIs128[str-3*p::*gfp]* X |  | 4E-F |
| PY5397 | *crh-1*(*tz2*) III; *str-3*p::*gfp* X |  | 4E-F |
| KHK1431 | PY5397 Ex[*srg-47*p::*crh-1*cDNA;*unc122*p::*dsRed*] line1 |  | 4F |
| KHK1432 | PY5397 Ex[*srg-47*p::*crh-1*cDNA;*unc122*p::*dsRed*] line2 |  | 4F |
| KHK1433 | PY5397 Ex[*srg-47*p::*crh-1*cDNA;*unc122*p::*dsRed*] line3 |  | 4F |
| CB1372 | *daf-7(e1372)* III | CGC | 5A, 5E |
| KHK1382 | *crh-1*(*tz2*); *daf-7(e1372)* III |  | 5A |
| KHK1441 | *daf-7(e1372)* III ; Ex[*flp-8*p::*gfp*] | crossed from KHK1401 | 5B, S2 |
| CB1385 | *daf-5*(*e1385*) II | CGC | 5C |
| GR1311 | *daf-3*(*mgDf90*) X | CGC | 5C |
| KHK1384 | *daf-5*(*e1385*) II; *crh-1*(*tz2*) III |  | 5C |
| KHK1383 | *crh-1*(*tz2*) III; *daf-3*(*mgDf90*) X |  | 5C |
| KHK1387 | *crh-1*(*tz2*) III; *Ex[srg-47*p::*daf-7*cDNA *unc-122*p::*dsRed]* line1 |  | 5D |
| KHK1388 | *crh-1*(*tz2*) III; *Ex[srg-47*p::*daf-7*cDNA *unc-122*p::*dsRed]* line2 |  | 5D |
| KHK1389 | *daf-7(e1372)* III; *Ex[srg-47*p::*daf-7*cDNA *unc-122*p::*dsRed]* line1 |  | 5E |
| KHK1390 | *daf-7(e1372)* III; *Ex[srg-47*p::*daf-7*cDNA *unc-122*p::*dsRed]* line2 |  | 5E |
| KHK1508 | *Ex[daf-7*p::*gfp unc-122*p::*dsRed]* line1 |  | 6B-D, S13 |
| KHK1509 | *Ex[daf-7*p::*gfp unc-122*p::*dsRed]* line2 |  | 6B, S13 |
| KHK1611 | *crh-1*(*tz2*) III; *Ex[daf-7*p::*gfp unc-122*p::*dsRed]* line1 |  | 6B, S13 |
| KHK1612 | *crh-1*(*tz2*) III; *Ex[daf-7*p::*gfp unc-122*p::*dsRed]* line2 |  | 6B, S13 |
| KHK1510 | *Ex[daf-7p(mutated at -2494bp)*::*gfp unc-122*p::*dsRed]* line1 |  | 6B, S13 |
| KHK1511 | *Ex[daf-7p(mutated at -2494bp)*::*gfp unc-122*p::*dsRed]* line2 |  | 6B, S13 |
| KHK1512 | *Ex[daf-7p(mutated at -2410bp)*::*gfp unc-122*p::*dsRed]* line1 |  | 6B, S13 |
| KHK1513 | *Ex[daf-7p(mutated at -2410bp)*:: *gfp unc-122*p::*dsRed]* line2 |  | 6B, S13 |
| KHK1514 | *Ex[daf-7p(mutated at -2381bp)*::*gfp unc-122*p::*dsRed]* line1 |  | 6B-D, S13 |
| KHK1515 | *Ex[daf-7p(mutated at -2381bp)*:: *gfp unc-122*p::*dsRed]* line2 |  | 6B, S13 |
| KHK1519 | *Ex[daf-7p(mutated at -2344bp)*::*gfp unc-122*p::*dsRed]* line1 |  | 6B, S13 |
| KHK1520 | *Ex[daf-7p(mutated at -2344bp)*:: *gfp unc-122*p::*dsRed]* line2 |  | 6B, S13 |
| KHK1521 | *Ex[daf-7p(mutated at -1476bp)*::*gfp unc-122*p::*dsRed]* line1 |  | 6B, S13 |
| KHK1522 | *Ex[daf-7p(mutated at -1476bp)*::*gfp unc-122*p::*dsRed]* line2 |  | 6B, S13 |
| KHK1641 | *Ex[daf-7p(mutated at -1436bp)*::*gfp unc-122*p::*dsRed]* line1 |  | 6B, S13 |
| KHK1642 | *Ex[daf-7p(mutated at -1436bp)*:: *gfp unc-122*p::*dsRed]* line2 |  | 6B, S13 |
| KHK1523 | *Ex[daf-7p(mutated at -1419bp)*::*gfp unc-122*p::*dsRed]* line1 |  | 6B, S13 |
| KHK1524 | *Ex[daf-7p(mutated at -1419bp)*:: *gfp unc-122*p::*dsRed]* line2 |  | 6B, S13 |
| KHK1525 | *Ex[daf-7p(mutated at -941bp)*::*gfp unc-122*p::*dsRed]* line1 |  | 6B, S13 |
| KHK1526 | *Ex[daf-7p(mutated at -941bp)*:: *gfp unc-122*p::*dsRed]* line2 |  | 6B, S13 |
| KHK1527 | *Ex[daf-7p(mutated at -383bp)*::*gfp unc-122*p::*dsRed]* line1 |  | 6B, S13 |
| KHK1528 | *Ex[daf-7p(mutated at -383bp)*:: *gfp unc-122*p::*dsRed]* line2 |  | 6B, S13 |
| KHK1705 | *crh-1*(*tz2*) III; *Ex[daf-7*p::*gfp unc-122*p::*dsRed]* line1 | outcrossed from KHK1508 | 6C-D |
| KHK1706 | *crh-1*(*tz2*) III; *Ex[daf-7p(mutated at -2381bp)*::*gfp unc-122*p::*dsRed]* line1 | outcrossed from KHK1514 | 6C-D |
|  | *gpa-4*p::*gfp(oyIs55)* |  | S11 |
| PY5416 | *crh-1*(*tz2*) III; *gpa-4*p::*gfp(oyIs55)* |  | S11 |
| CF1038 | *daf-16(mu86)* I | CGC | S12 |
| KHK1442 | *daf-16(mu86)* I; *crh-1*(*tz2*) III |  | S12 |
| KHK2187 | *Ex[nhr-246*p::*gfp unc-122*p::*dsRed]* |  | S5 |
| KHK2188 | *crh-1*(*tz2*) III; *Ex[nhr-246*p::*gfp unc-122*p::*dsRed* |  | S5 |

^a^CGC – *Caenorhabditis* Genetics Center
