# Supplementary material for: CREB mediates the C. elegans dauer polyphenism through direct and cell-autonomous regulation of TGF-β expression
Source: PLoS Genet. 2021 Jul 14;17(7):e1009678. doi: 10.1371/journal.pgen.1009678 (PMC8312985; doi:10.1371/journal.pgen.1009678)
Supplement: S2 Table — (DOCX) [file pgen.1009678.s015.docx]

**S2 Table. List of primers and probes used in *daf-7* related work.**

| Name of primer | Sequence |
| --- | --- |
| *sph daf-7* promoter forward primer | AAA AAA GCA TGC GGA AGC TTC GGC AAC TA |
| *kpn daf-7* promoter backward primer | AAA AAA GCA TGC GGA AGC TTC GGC AAC TA |
| -2943 forward primer | TTG GCA TAA TCA GAA GAT ATT GGG GGA GC |
| -2943 backward primer | TAA TTG GAC GCT TGC GTG CTC CCC CAA TA |
| -2410 forward primer | TAA TTG GAC GCT TGC GTG CTC CCC CAA TA |
| -2410 backward primer | AAT CCG CAT TGA AAA AAG TAC CCT AAT TGG |
| -2381 forward primer | GCT TTC AGC TTA AGG GGT GTT CTT TTC AAC |
| -2381 backward primer | GTT GAA AAG AAC ACC CCT TAA GCT GAA AGC |
| -2344 forward primer | AGA ATT ATT CAC AGG GGT TTA CTC CTG AAA |
| -2344 backward primer | TTT CAG GAG TAA ACC CCT GTG AAT AAT TCT |
| -1476 forward primer | CTA TGA AGT TGC GGG GGT TTG CCG AGA AGT |
| -1476 backward primer | ACT TCT CGG CAA ACC CCC GCA ACT TCA TAG |
| -1436 forward primer | TGGGAGTTCCACTGGGGCGATGGAATAGGA |
| -1436 backward primer | TCCTATTCCATCGCCCCAGTGGAACTCCCA |
| -1419 forward primer | TTTAAAACCTCAAGGGGTGCTTAAACGGGT |
| -1419 backward primer | ACCCGTTTAAGCACCCCTTGAGGTTTTAAA |
| -941 forward primer | TTTTAGATGAAGAGGGGCCCCCGATGAAGA |
| -941 backward primer | TCTTCATCGGGGGCCCCTCTTCATCTAAAA |
| -383 forward primer | CCCCCGATGAAGAGGGGCGGAATCCGGCAC |
| -383 backward primer | GTGCCGGATTCCGCCCCTCTTCATCGGGGG |
| *daf-7* probe 3x  (biotin labeled and unlabeded) | attaggGTACGTACgtcaatattaggGTACGTACgtcaatattaggGTACGTACgtcaat |
| Self-mutant competitor of *daf-7* probe1 | attaggGTATTTTTttcaatattaggGTATTTTTttcaatattaggGTATTTTTttcaat |
| Self-mutant competitor of *daf-7* probe2 | attaggTGACGTCAgtcaatattaggTGACGTCAgtcaatattaggTGACGTCAgtcaat |
| Reference (conventional CRE) competitor of *daf-7* probe | attaggTGACGTCAgtcaatattaggTGACGTCAgtcaatattaggTGACGTCAgtcaat |
